# Meaning and purpose in Huntington’s disease: a longitudinal study of its impact on quality of life

**DOI:** 10.1002/acn3.51424

**Published:** 2021-07-20

**Authors:** Leonard L. Sokol, Jonathan P. Troost, Benzi M. Kluger, Allison J. Applebaum, Jane S. Paulsen, Danny Bega, Samuel Frank, Joshua M. Hauser, Nicholas R. Boileau, Colin A. Depp, David Cella, Noelle E. Carlozzi

**Affiliations:** ^1^ The Ken and Ruth Davee Department of Neurology Northwestern University Feinberg School of Medicine Chicago Illinois USA; ^2^ Center for Bioethics and Humanities McGaw Bioethics Scholars Program Northwestern University Feinberg School of Medicine Chicago Illinois USA; ^3^ Michigan Institute for Clinical and Health Research University of Michigan Ann Arbor Michigan USA; ^4^ Departments of Neurology and Medicine University of Rochester Medical Center Rochester New York USA; ^5^ Department of Psychiatry & Behavioral Sciences Memorial Sloan Kettering Cancer Center New York New York USA; ^6^ Department of Neurology University of Wisconsin‐Madison Madison Wisconsin USA; ^7^ Department of Neurology Beth Israel Deaconess Medical Center Harvard Medical School Boston Massachusetts USA; ^8^ Department of Medicine Feinberg School of Medicine and Palliative Care Service Jesse Brown VA Medical Center Chicago Illinois USA; ^9^ Department of Physical Medicine and Rehabilitation University of Michigan Ann Arbor Michigan USA; ^10^ Department of Psychiatry University of California San Diego California USA; ^11^ Department of Medical Social Sciences Northwestern University Feinberg School of Medicine Chicago Illinois USA

## Abstract

**Objective:**

Previous work in Huntington’s disease (HD) has shown that a sense of meaning and purpose (M&P) is positively associated with positive affect and well‐being (PAW); however, it was unknown whether HD‐validated patient‐reported outcomes (PROs) influence this association and how M&P impacts PROs in the future. Our study was designed to examine if HD‐validated PROs moderate the relationship between M&P and PAW and to evaluate if baseline M&P predicts 12‐ and 24‐month changes in HD‐validated PROs.

**Methods:**

This was a longitudinal, multicenter study to develop several PROs (e.g., specific for the physical, emotional, cognitive, and social domains) for people with HD (HDQLIFE). The sample consisted of 322 people with HD (*n* = 50 prodromal, *n* = 171 early‐stage manifest, and *n* = 101 late‐stage manifest HD). A single, multivariate linear mixed‐effects model was performed with PAW as the outcome predicted by main effects for M&P and several moderators (i.e., an HD‐validated PRO) and interactions between M&P and a given PRO. Linear‐mixed models were also used to assess if baseline M&P predicted HD‐validated PROs at 12 and 24 months.

**Results:**

Higher M&P was positively associated with higher PAW regardless of the magnitude of symptom burden, as represented by HD‐validated PROs, and independent of disease stage. In our primary analysis, baseline M&P predicted increased PAW and decreased depression, anxiety, anger, emotional/behavioral disruptions, and cognitive decline at 12 and 24 months across all disease stages.

**Interpretation:**

These findings parallel those seen in the oncology population and have implications for adapting and developing psychotherapeutic and palliative HD interventions.

## Introduction

Huntington’s disease (HD) is an autosomal dominant, progressive neurodegenerative disease. There is currently no available cure or disease‐modifying agent.^1^ HD affects approximately 30,000 people in the United States, with another 150,000 living at‐risk.^2^ Symptoms typically manifest between ages 30 and 50 with the triad of motor, cognitive, and emotional/behavioral impairments. There is a marked scarcity of evidence‐based treatments.^3^ Suicide is a leading cause of death.^4^

As part of a national initiative to improve the measurement of health‐related quality of life (HRQOL) in HD, several stakeholders (e.g., clinicians, informal care partners, people with the HD genetic mutation) convened and identified several symptoms significant within the HD triad (i.e., motor, cognitive, and emotional/behavioral disturbances).^5^ This resulted in the formation of several HD‐validated patient‐reported outcomes (PROs). These PROs have subsequently undergone rigorous psychometric and clinometric testing among people with HD genetic mutation across various disease stages.^6^ The PROs include outcomes, such as stigma, anxiety, anger, satisfaction with social roles, chorea, speech, and swallowing difficulties, concerns with death and dying, end of life planning, meaning and purpose, positive affect and well‐being, cognition, and emotional/behavioral disruptions.

Despite the multitude of PROs, the non‐motor ones (e.g., cognition or depression) seem to be more potent contributors to HRQOL scores than do the motor impairments, and perhaps more so in the earlier disease stages.^7^, ^8^, ^9^ Additionally, there is a high prevalence of spiritual and existential distress (hopelessness, concern with death and dying, suicidality),^10^, ^11^ which is more salient than in other neurological diseases.^12^ However, there are no evidence‐based palliative care models or psychotherapeutic interventions that can ameliorate these distressing symptoms among people with the HD gene mutation.^9^, ^13^, ^14^

Work within oncology has suggested that a sense of meaning and purpose may buffer against depression and suicidality.^15^, ^16^, ^17^, ^18^, ^19^ Work within palliative care and psycho‐oncology have further substantiated these observations.^20^, ^21^, ^22^, ^23^, ^24^, ^25^, ^26^ Meaning‐centered interventions that allow people with advanced cancer to connect to various forms of meaning are associated with reductions in spiritual and existential distress and improvements in HRQOL.^27^, ^28^, ^29^, ^30^, ^31^ Previous reports among people with the HD genetic mutation have suggested that the most robust positive association with meaning and purpose (M&P) is positive affect and well‐being (PAW).^32^ However, it is unclear if this relationship is maintained when considering the magnitude of symptom burden as conceptualized with HD‐validated PROs (e.g., chorea, difficulties with speech and swallowing, depression, etcetera). Furthermore, it was unknown whether M&P may predict longitudinal changes in HD‐validated PROs.

Therefore, our overall goal of the study was to establish whether “meaning” represents a rational therapeutic target for intervention development to alleviate spiritual and existential suffering among people with HD gene mutation. Our objectives were twofold. Our first objective was to determine if an HD PRO, M&P, as embodied by the HDQLIFE M&P questionnaire,^11^ will remain positively associated with joy, life contentment, and happiness, as measured by the NeuroQOL PAW questionnaire,^33^ despite the burden of a variety of physical (e.g., chorea), emotional (e.g., depression), or social (e.g., ability to participate in activities) symptoms, as characterized by several NeuroQOL/PROMIS and HDQLIFE validated PROs for this population. We hypothesized that regardless of the disease stage, people with either premanifest or manifest HD and higher M&P would express higher levels of PAW, even in the face of severe symptomatology (Hypothesis 1). Our second objective was to ascertain if M&P at baseline predicted longitudinal HRQOL outcomes among a cohort of people with pre‐, early‐, and late‐stage manifest HD at 12 and 24 months. We hypothesized that baseline M&P would predict future improvements in PAW and reductions in negative emotional PROs (Hypothesis 2).

## Methods

### Participants

This analysis included 322 people with the gene mutation for HD (*n* = 50 prodromal, *n* = 171 early‐stage manifest, and *n* = 101 late‐stage manifest HD). Participants were part of a larger study, conducted between 2012 and 2016, designed to develop and validate new measures of HRQOL; full details of that study are reported elsewhere.^6^ Eligibility criteria included a positive gene test and/or a clinical diagnosis of HD, ≥18 years of age, and the ability to provide informed consent (cognitive status was confirmed using a standard assessment when warranted^34^). Study participants were recruited through established movement disorders clinics, support groups, nursing homes with HD‐specific units, the National Research Roster for Huntington’s Disease, existing research registries, online medical record data capture systems,^35^ and through articles/advertisements targeting the HD community. A portion of the sample was collected in conjunction with the Predict‐HD study, a longitudinal, global cohort study.^36^

### Participant characterization and clinician‐administered measure of functioning

The Problem Behaviors Assessment is a clinician‐rated tool based on a semi‐structured interview; one of the domains is suicidality (PBA‐s). A score of “0” means no symptoms and “4” means symptoms are occurring daily. Eighty‐four percent of our cohort had a PBA‐s score of 0; the remaining were >0. The Total Functional Capacity (TFC) from the Unified Huntington’s Disease Ratings Scales (UHDRS)^37^ was used to characterize people's functional capacity with the HD gene mutation in this sample. TFC provides an assessment of an individual’s ability to work, manage finances, do chores, live independently, perform daily living activities, and determine the HD stage for those with manifest HD. Total scores range from 0 to 13, with higher scores indicating better ability. Early‐stage manifest HD was defined as TFC scores of 7–13, and late‐stage manifest HD was defined as TFC scores of 0–6.^38^ In addition, the final question on the UHDRS was used to determine manifest versus premanifest HD. Specifically, this question asks the clinician to rate their confidence from 1 (0% confidence) to 4 (>99% confidence) that the participant has motor manifest HD. Participants who received a rating of 3 or less were classified as having prodromal HD.

### PRO administration formats and scoring

All PROs, except M&P and Anger, were administered as computer adaptive tests (CATs) followed by the fixed short‐forms (SFs); M&P and Anger were administered as SFs only. We examined scores from the CAT administrations for all PROs when available (all PROs except for M&P and Anger). The resulting T‐scores (*M* = 50; SD = 10) are relative to the development population and indicate more of that domain being measured (i.e., higher M&P scores indicate a better sense of purpose—better HRQOL, whereas higher depression scores indicate more sadness—worse HRQOL). The degree of symptom severity was defined based on one standard deviation above and below, which was used to generate groups of high and low people, respectively, on a given PRO.^39^

### PRO measures

HDQLIFE M&P^6^, ^11^ assesses an individual’s beliefs about why we do the things we do and make the most out of the time we have; data support its reliability, validity, and responsiveness in HD.^6^, ^11^, ^40^

Neuro‐QoL Depression^41^ assesses perceptions of sadness and helplessness; data support its reliability, validity, and responsiveness in HD.^6^, ^42^, ^43^

Neuro‐QoL Anxiety^41^ assesses feelings of nervousness and fear; data support its reliability, validity, and responsiveness in HD.^6^, ^42^, ^43^

PROMIS Anger^44^, ^45^, ^46^ assesses feelings of frustration and anger; data support its reliability, validity, and responsiveness in HD.^6^, ^42^, ^43^

Neuro‐QoL PAW^41^ assesses feelings of happiness, enjoyment, and contentment; data support its reliability, validity, and responsiveness in HD.^6^, ^42^, ^43^

Neuro‐QoL Stigma^41^ assesses perceptions of discrimination toward an individual; data support its reliability, validity, and responsiveness in HD.^6^, ^42^, ^43^

Neuro‐QoL Satisfaction with Social Roles and Activities (SRA)^41^ assesses an individual’s perceptions of satisfaction with social roles and activities.; data support its reliability, validity, and responsiveness in HD.^6^, ^42^, ^43^

HDQLIFE Chorea^6^, ^47^ assesses the impact that chorea (i.e., the dance‐like movements characteristic of HD) has on physical activity and participation; data support its reliability, validity, and responsiveness in HD.^6^, ^47^, ^48^, ^49^

HDQLIFE Speech Difficulties^6^, ^50^ assesses how difficulty with oral expression, language production, and articulation affects communication and well‐being; data support its reliability, validity, and responsiveness in HD.^6^, ^48^, ^49^, ^50^

HDQLIFE Swallowing Difficulties^6^, ^50^ assesses how swallowing and choking problems impact well‐being and eating; data support its reliability, validity, and responsiveness in HD.^6^, ^48^, ^49^, ^50^

HDQLIFE Concern with Death and Dying^6^, ^11^ assesses a person’s thoughts about death and dying^6^, ^11^; data support its reliability, validity, and responsiveness in HD.^6^, ^11^, ^40^

HDQLIFE End of Life Planning^40^, ^51^ assesses a person’s wishes and preparation about future medical care, including topics related to institutionalization, hospice, and environments desired near death; data support its reliability, validity, and responsiveness in HD.^51^

NeuroQOL Cognitive Function^52^ assesses a person’s perceived abilities in memory, attention, and other executive functions; data support its reliability, validity, and responsiveness in HD.^43^, ^52^, ^53^

NeuroQOL Emotional and Behavioral Dyscontrol^5^, ^43^, ^47^ assess a person’s impulsivity, lability, and irritability; datasupport its reliability, validity, and responsiveness in HD.

### Procedures

Participants completed assessments at baseline, 12 and 24 months. All study visits were approximately 2 h in duration and involved completing an in‐person assessment and an online survey (administered through Assessment Center^SM^ ^54^). All participants provided informed consent at the baseline visit. At 12 and 24 months, participants who were unable or unwilling to be seen in person were provided a telephone interview option. Local Institutional Review Boards approved data collection.

### Statistical analysis plan

PAW at each visit was modeled using linear mixed‐effects repeated measures models with a compound symmetry covariance structure (determined based on the mode of fit). A single model was performed with PAW as the outcome predicted by main effects for M&P and PRO moderators (i.e., depression, anxiety, anger, social participation, chorea, speech, swallow, concern with death and dying, end of life planning, suicidal behaviors, cognition, emotional/behavioral disruptions, and UHDRS/TFC); that is, an interaction between M&P and a moderator—while adjusted for other PROs and other interactions. All variables were treated as continuous except suicidality, which was dichotomized as any versus none. All moderators were tested in a multivariate linear mixed effect model (i.e., depression, anxiety, anger, satisfaction with social roles, chorea, speech, swallowing, concern with death & dying, stigma, end of life planning, suicidal behaviors, cognition, and emotional/behavioral disruptions). The slopes for the relationship between M&P and PAW were reported and plotted for low and high levels of each moderator (low = *t*‐score 40; high = *t*‐score 60; for suicidality low = none; high = any) to aid in interpreting interactions. A significant *p*‐value (<0.05) was interpreted as evidence of effect modification in the relationship between M&P and PAW; a non‐significant *p*‐value (≥0.05) suggested that the relationship between M&P and PAW was the same across levels of the moderator.

Linear‐mixed models were also used to assess if baseline M&P predicted PAW at follow‐up visits at 12 and 24 months after controlling for baseline PAW. An analogous approach was used to model for PROs and clinician‐administered levels of functioning. We accounted for multiple comparisons by reporting false‐discovery rate‐adjusted *p*‐values using linear step‐up.^55^ Analyses were performed in SAS V9.4 (SAS Institute Inc., Cary, NC, USA).

## Results

Data on the number of people eligible, their clinical/demographic data, who completed follow‐up, study attrition, and how missing data was accounted for may be found elsewhere as part of the more extensive HDQLIFE study.^6^ Descriptive characteristics for participants in this analysis are shown in Table 1.

**Table 1:** Descriptive characteristics.

Characteristic	Prodromal (*n* = 50)	Early (*n* = 171)	Late (*n* = 101)	All (*n* = 322)	*p*‐value
Age (years)					<0.0001
Mean (SD)	43.4 (11.29)	51.6 (12.62)	55.5 (11.71)	51.6 (12.72)	
*N*	50	171	101	322	
Gender, *n* (%)					0.5942
Female	23 (46)	82 (48)	42 (42)	147 (46)	
Male	27 (54)	89 (52)	59 (58)	175 (54)	
Ethnicity, *n* (%)					0.6048
Not Hispanic or Latino	48 (96)	159 (93)	98 (97)	305 (95)	
Hispanic of Latino	1 (2)	7 (4)	1 (1)	9 (3)	
Not provided	1 (2)	5 (3)	2 (2)	8 (2)	
Race, *n* (%)					0.0032
Caucasian	48 (96)	164 (96)	93 (92)	305 (95)	
African American	0 (0)	2 (1)	8 (8)	10 (3)	
Other	1 (2)	5 (3)	0 (0)	6 (2)	
Unknown	1 (2)	0 (0)	0 (0)	1 (0)	
Education (years)					0.0111
Mean (SD)	15.7 (2.93)	14.6 (2.70)	14.2 (2.53)	14.7 (2.72)	
*N*	50	164	98	312	
Marital status, *n* (%)					0.1216
Single, never married	5 (10)	28 (16)	11 (11)	44 (14)	
Married	36 (72)	88 (51)	63 (62)	187 (58)	
Separated/divorced	8 (16)	42 (25)	23 (23)	73 (23)	
Living with partner	1 (2)	7 (4)	0 (0)	8 (2)	
Widowed	0 (0)	6 (4)	4 (4)	10 (3)	
CAG repeats					0.0115
Mean (SD)	41.9 (2.31)	43.2 (3.98)	44.8 (7.21)	43.3 (4.71)	
Domain scores, mean (SD)					
Positive affect & well‐being	55.1 (8.74)	54.9 (8.51)	54.3 (8.46)	54.8 (8.51)	0.7249
Meaning & purpose	49.0 (10.02)	50.3 (9.75)	48.7 (8.32)	49.6 (9.38)	0.1904
Depression	49.4 (9.98)	51.3 (10.80)	51.3 (11.08)	51.0 (10.75)	0.5892
Anxiety	52.8 (9.68)	53.4 (10.05)	54.1 (11.35)	53.5 (10.39)	0.8580
Anger	48.3 (12.11)	48.4 (12.32)	47.1 (12.75)	48.0 (12.40)	0.7638
Social participation	50.0 (8.29)	47.1 (7.87)	42.8 (7.97)	46.3 (8.33)	<0.001
Chorea	42.9 (6.81)	52.9 (7.32)	57.4 (7.29)	52.8 (8.60)	<0.001
Speech	45.3 (6.84)	50.7 (7.57)	55.2 (7.83)	51.3 (8.19)	<0.001
Swallow	46.1 (7.16)	51.6 (8.14)	56.0 (7.64)	52.1 (8.45)	<0.001
Concern with death and dying	50.0 (9.14)	50.6 (9.58)	49.8 (11.02)	50.3 (9.96)	0.7139
Stigma	46.1 (7.66)	51.7 (8.00)	53.3 (9.67)	51.3 (8.78)	<0.001
End of life planning	47.4 (8.23)	49.8 (9.63)	53.2 (10.18)	50.5 (9.78)	<0.001
Suicidal behaviors	0.6 (2.23)	0.4 (1.48)	0.4 (1.45)	0.4 (1.61)	0.9277
Cognition	44.4 (10.33)	38.3 (8.94)	28.6 (7.83)	36.3 (10.43)	<0.001
Emotional/behavioral disruptions	46.2 (11.06)	47.4 (10.40)	46.9 (11.63)	47.0 (10.86)	0.6064
UHDRS/TFC	12.2 (1.58)	9.9 (1.96)	3.9 (1.80)	8.4 (3.65)	<0.001

### Hypothesis 1. People with the HD gene mutation and high levels of M&P would report high levels of joy, contentment, and happiness even in the face of high levels of symptoms

**Figure 1:**
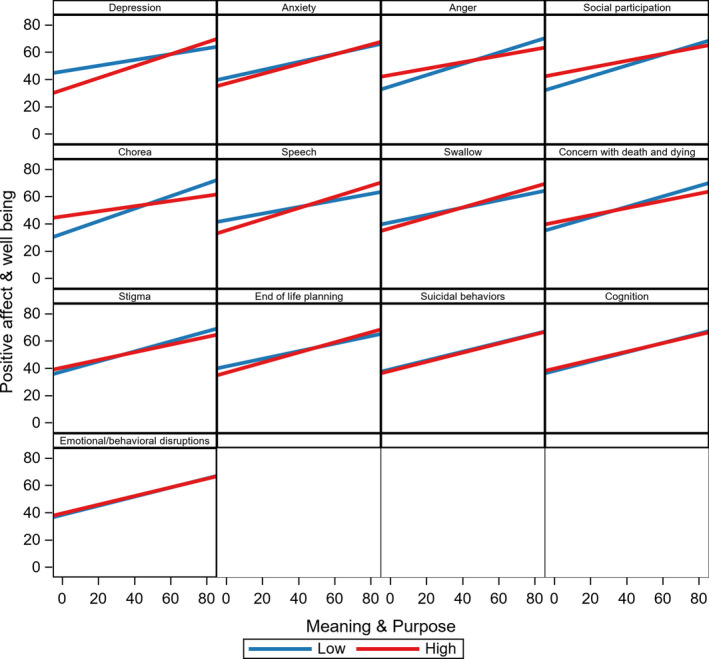
The impact of meaning & purpose on positive affect & well‐being by moderator levels based on estimates in Table 2.

In a previous bivariate analysis, M&P was moderately correlated with PAW (*r* = 0.63, *p* < 0.01).^32^ Table 2 includes the results of our linear‐mixed model. The impact of M&P on PAW was moderated by only two other PROs (i.e., depression and chorea). Specifically, there was a positive interaction with depression (*p* = 0.0258), indicating a stronger relationship among those with higher depression, and negative interaction with chorea (*p* = 0.0048), indicating a weaker relationship among those with higher chorea. Model estimates were used to calculate strata‐specific slopes for those with low and high levels of each symptom; slopes for the relationship between M&P and PAW are shown in Table 3. For depression, there was a 0.21‐point *t*‐score increase in PAW per 1 point increase in M&P among those with low depression; among those with high depression, there was a 0.44‐point *t*‐score increase. For low and high chorea, the M&P slopes were 0.46 and 0.19, respectively, but both were statistically significant. These relationships are plotted as line graphs in Figure 1. For all other PROs in our analysis, the relationship between M&P and PAW remained the same for those with high or low symptoms of a given PRO. There were no significant subgroup differences, either by gender or disease stage.

**Table 2:** Linear‐mixed effects model of positive affect & well‐being.

Variable	Main effects	*p*‐value	Interactions (with M&P)	
	Beta [95% CI]		Beta [95% CI]	*p*‐value
Intercept	25.082	–	–	–
Meaning & purpose	0.814 [−0.112, 1.740]	0.0847	–	–
Depression	−0.685 [−1.181, −0.189]	0.0069	0.011 [0.001, 0.021]	0.0258
Anxiety	−0.210 [−0.772, 0.352]	0.4632	0.003 [−0.008, 0.014]	0.5417
Anger	0.416 [−0.067, 0.899]	0.0912	−0.009 [−0.019, 0.001]	0.0756
Social participation	0.472 [0.089, 0.856]	0.0159	−0.008 [−0.015, 0.000]	0.0502
Chorea	0.624 [0.148, 1.101]	0.0104	−0.014 [−0.023, −0.004]	0.0048
Speech	−0.375 [−0.910, 0.159]	0.1684	0.008 [−0.002, 0.019]	0.1117
Swallow	−0.212 [−0.667, 0.242]	0.3594	0.005 [−0.004, 0.014]	0.2460
Concern with death and dying	0.186 [−0.148, 0.520]	0.2737	−0.006 [−0.013, 0.001]	0.0836
Stigma	0.150 [−0.320, 0.619]	0.5314	−0.005 [−0.014, 0.005]	0.3517
End of life planning	−0.221 [−0.513, 0.071]	0.1375	0.005 [−0.001, 0.010]	0.1111
Suicidal behaviors	−1.044 [−9.318, 7.230]	0.8043	0.008 [−0.169, 0.186]	0.9272
Cognition	0.084 [−0.263, 0.431]	0.6348	−0.001 [−0.008, 0.005]	0.6663
Emotional/behavioral disruptions	0.055 [−0.501, 0.611]	0.8462	−0.001 [−0.012, 0.010]	0.8768

**Table 3:** The impact of meaning & purpose on positive affect & well‐being by moderator levels based on estimates in Table 2.

Variable	Levels of moderator			
	Low	*p*‐value	High	*p*‐value
	M&P Beta [95% CI]		M&P Beta [95% CI]	
Depression	0.21 [0.07, 0.36]	0.0041	0.44 [0.28, 0.60]	<0.0001
Anxiety	0.29 [0.11, 0.47]	0.0018	0.36 [0.23, 0.49]	<0.0001
Anger	0.41 [0.26, 0.57]	<0.0001	0.24 [0.09, 0.39]	0.0022
Social participation	0.40 [0.27, 0.54]	<0.0001	0.25 [0.11, 0.39]	0.0007
Chorea	0.46 [0.31, 0.61]	<0.0001	0.19 [0.04, 0.34]	0.0136
Speech	0.24 [0.11, 0.38]	0.0004	0.41 [0.23, 0.58]	<0.0001
Swallow	0.27 [0.13, 0.42]	0.0002	0.38 [0.23, 0.53]	<0.0001
Concern with death and dying	0.38 [0.25, 0.52]	<0.0001	0.27 [0.13, 0.40]	0.0001
Stigma	0.37 [0.23, 0.52]	<0.0001	0.28 [0.12, 0.44]	0.0005
End of life planning	0.28 [0.15, 0.40]	<0.0001	0.37 [0.24, 0.51]	<0.0001
Suicidal behaviors	0.32 [0.20, 0.44]	<0.0001	0.33 [0.14, 0.52]	0.0006
Cognition	0.34 [0.26, 0.42]	<0.0001	0.31 [0.14, 0.48]	0.0004
Emotional/behavioral disruptions	0.33 [0.20, 0.47]	<0.0001	0.32 [0.13, 0.50]	0.0008

### Hypothesis 2. Baseline M&P for people with the HD gene mutation would predict an increased PAW and decreased negative emotional PROs at 12 and 24 months

The predictors are baseline M&P and baseline levels of the outcome. Scores from baseline, 12, and 24 months are modeled—after adjusting for what the levels of each of the outcomes were at baseline (Table 4). M&P at baseline was associated with increased PAW (Beta 95% CI = 0.15 [0.06, 0.24], p = 0.0011), decreased depression (Beta 95% CI = −0.16 [−0.27, −0.06], *p* = 0.0030), decreased anxiety (Beta 95% CI = −0.12 [−0.22, −0.02], *p* = 0.0202), decreased anger (Beta 95% CI = −0.20 [−0.33, −0.08], *p* = 0.0011), decreased emotional/behavioral disruptions (Beta 95% CI = −0.19 [−0.28, −0.09], *p* = 0.0001), and slowed cognitive decline (Beta 95% CI = 0.10 [0.01, 0.19], *p* = 0.0329), at both 12 and 24 months. The strongest relationship was seen with anger: a 1 *t*‐score increase in M&P at baseline is associated with a 0.2 *t*‐score decrease in anger at follow‐up (regardless of where anger was at baseline). Our analysis that adjusted for multiple comparisons maintained that PAW, depression, anger, and emotional/behavioral disruptions were still significant; however, anxiety and cognition were not. Additionally, no evidence of subgroup variation was seen, except a positive association between baseline M&P and follow‐up social participation, as assessed through NeuroQOL SRA, among the prodromal group (Beta 95% CI = 0.12 [0.01–0.23], *p* = 0.04) but was not found in other groups.

**Table 4:** Linear‐mixed effects models of baseline M&P on symptoms at 12 and 24 months. (Suicidal behaviors dichotomized and analyzed using binary logit model).^1^

Outcome (assessed at t1 & t2)	Baseline M&P	*p*‐value	Baseline symptom	
	Beta and 95%		Beta and 95%	*p*‐value
Positive affect & well being	0.15 [0.06, 0.24]	0.0011	0.49 [0.38, 0.59]	<0.0001
Depression	−0.16 [−0.27, −0.06]	0.0030	0.54 [0.44, 0.64]	<0.0001
Anxiety	−0.12 [−0.22, −0.02]	0.0202	0.65 [0.55, 0.74]	<0.0001
Anger	−0.20 [−0.33, −0.08]	0.0011	0.52 [0.43, 0.62]	<0.0001
Social participation	0.01 [−0.07, 0.09]	0.7156	0.45 [0.36, 0.54]	<0.0001
Chorea	−0.03 [−0.08, 0.03]	0.3522	0.76 [0.70, 0.82]	<0.0001
Speech	−0.01 [−0.07, 0.05]	0.8103	0.73 [0.66, 0.80]	<0.0001
Swallow	0.01 [−0.06, 0.07]	0.7723	0.78 [0.71, 0.85]	<0.0001
Concern with death and dying	−0.03 [−0.12, 0.06]	0.5022	0.55 [0.46, 0.64]	<0.0001
Stigma	−0.00 [−0.08, 0.07]	0.9006	0.64 [0.55, 0.72]	<0.0001
End of life planning	0.00 [−0.07, 0.08]	0.9270	0.79 [0.72, 0.86]	<0.0001
Suicidal behaviors	−0.02 [−0.05, 0.02]	0.3550	2.10 [1.36, 2.83]	<0.0001
Cognition	0.10 [0.01, 0.19]	0.0329	0.64 [0.56, 0.72]	<0.0001
Emotional/behavioral disruptions	−0.19 [−0.28, −0.09]	0.0001	0.52 [0.43, 0.60]	<0.0001
UHDRS/TFC	0.00 [−0.02, 0.02]	0.6646	0.86 [0.81, 0.92]	<0.0001

^1^ Note: Each line represents an independent linear mixed‐effects model with two covariates: (1) M&P at baseline and (2) levels of outcome domain at baseline.

## Discussion

Our results support two key relationships between M&P and HRQOL. First, people with the HD gene mutation who express higher M&P exhibit higher contentment and joy in their lives, independent of the degree of symptom severity experienced. Interestingly, the impact that M&P has on PAW may be more potent even in the face of high depression, suggesting a distinct mechanism by which M&P operates to influence PAW. Namely, the magnitude that M&P has on PAW is greater at high depression than with low depression, suggesting that screenings indicative of high depressive burden could prompt clinicians to refer people with the HD genetic mutation for M&P interventions, which may still significantly influence joy, contentment, and happiness with life. However, the magnitude of M&P and PAW may be mildly attenuated in the face of severe chorea. Therefore, other pharmaco‐ or physio‐ therapies may be necessary to address severe chorea as it may attenuate this relationship. Second, in our primary analysis, baseline M&P was associated with increased PAW and decreased depression, anxiety, impulsivity, cognitive decline, and anger at 12 and 24 months.

Our findings support our first hypothesis that severe non‐motor or motor symptomatology does not negate the positive relationship between M&P and PAW and extends a large body of work in psycho‐oncology. In particular, our data are analogous and extend previous studies from people with advanced cancer, who are more likely to rate high levels of life satisfaction, even in the face of severe symptoms, when they concurrently report high levels of spiritual well‐being.^26^ Second, these findings also recapitulate that metaphysical factors beyond clinical depression or anxiety influence PAW, mostly since M&P was undeterred by those moderators. As the most robust correlation in HD to PAW is M&P,^32^ clinicians who may conflate existential suffering (i.e., low M&P) with clinical depression might miss an opportunity to intervene using a tailored approach (e.g., integration with chaplaincy). Indeed, our findings build upon data from other serious illnesses, which also observed that factors beyond depression influence negative emotional states, such as hastened death.^23^

Data addressing our second hypothesis support that baseline M&P predicts future HRQOL, especially the emotional and cognitive domains, suggesting future investigation areas.

First, while some may consider M&P to be a stable trait since findings suggest that M&P does not change across the different HD stages,^40^ interventions have successfully influenced M&P and other reputed state traits.^56^ Supporting this claim is that our results follow previous M&P interventions for the advanced cancer population that has shown positive emotional health changes^27^, ^28^, ^29^, ^30^ and, therefore, serve as an additional impetus to adapt an M&P intervention to HD.

Second, while the relationship of M&P and cognition did not hold after accounting for multiple comparisons, it is noteworthy that a seminal observational study within Alzheimer’s and other related dementias^57^ demonstrated that purpose in life was strongly associated with a lower incidence of Alzheimer’s. A dose–response relationship was also noted, such that a person who scored in the 90th percentile was 2.4 times more likely not to develop dementia as compared to a person in the 10th percentile—and this relationship was even maintained in the face of accounting for depression, chronic diseases, personality factors, and other demographic data. Further, higher purpose in life was associated with a lower risk for mild cognitive impairment development. Exploration of this association deserves further evaluation in HD, especially since around 80% suffer from mild cognitive impairment when motor symptoms manifest.^58^

Third, end‐of‐life planning,^59^ as represented by HDQLIFE End of Life Planning, is positively associated with M&P.^51^ Indeed, previous M&P interventions have also incorporated and positively influenced end‐of‐life planning.^30^ The inclusion of this practice to a future, adapted M&P intervention may also be warranted for this cohort, especially given a recent multicenter study of 503 people with HD that demonstrated the remarkably low prevalence of advance directives (38.2%), conversations about death, and dying with loved ones (10.5%), and deciding on a place to die (10.7%).^60^

Our analysis is not without limitations. First, we cannot determine causality because there was no experimental condition associated with the observational data. For example, might high positive affect give rise to high M&P?^61^, ^62^ Second, there are various instruments to measure the existential ideas within M&P.^63^ HDQLIFE represents one. However, our findings require verification with additional instrumentation to explore the longitudinal relationship between M&P and our PROs. Third, our models do not consider psychoactive medications, PT/OT utilization, or other exogenous factors, which may bias our analysis.

Despite these limitations, our findings are a compelling first step toward understanding the primary mechanism behind M&P and how it influences HRQOL in people with the HD gene mutation. Notably, M&P influences well‐known suicide risk factors for this population (anger, impulsivity, depression, and anxiety).^64^ Indeed, the SI rates in HD are much higher than other neurological diseases^12^, and some reports indicate that the highest suicide rates are before diagnosis (not immediately after) and in stage 2 (e.g., early‐stage manifest), as independence is lost.^64^ Models of suicidality suggest that impulsivity may be a necessary but not a sufficient factor in suicide attempts.^65^ Thus, a sense of M&P may serve as a resiliency factor for suicide in people with the HD gene mutation in that it can impact factors associated with suicidal ideation (e.g., depression, anxiety) as well as suicidal behaviors (e.g., impulsivity and anger).^66^

In conclusion, this study provides a generalization on the value of M&P to people with the HD gene mutation, and future efforts are warranted to adapt and develop meaning‐ and palliative‐ centered interventions to this population.^13^

## Conflict of Interest

The authors report no potential conflicts of interest related to the research covered in the article.

## Authors’ Contributions

All authors contributed to the conception, organization, execution of the project, and revising and critiquing the manuscript for important intellectual content.

1. Research project: A. Conception, B. Organization, C. Execution;

2. Statistical Analysis: A. Design, B. Execution, C. Review, and Critique;

3. Manuscript Preparation: A. Writing of the first draft, B. Review, and Critique.

LLS: 1A, 1B, 1C, 2A, 2C, 3A, 3B.

JT: 1B, 1C, 2A, 2B, 2C, 3B.

NC: 1A, 1B, 1C, 2A, 2B, 2C, 3B.

B.M.K, A.J.A., J.S.P., D.B., S.F., J.M.H., N.R.B., C.A.D., D.C.: 1A, 1B, 1C, 2C, 3B.

## Disclosures

Dr Sokol is an ad‐hoc consultant for Tikvah for Parkinson in the range of $0–$499, ad‐hoc consultant for the American Film Institute on end‐of‐life care/palliative care in the enrage of $500–$999; and receives financial support from the Northwestern PSTP Program in Neurology as well as the R25 NCI 2R25CA190169. Dr Troost has research funding through the University of Michigan with Complexa Inc, Retrophin Inc, and Goldfinch Bio, and the University of Michigan with Vertex Pharmaceuticals and Pfizer Inc. Dr Kluger received research grant support from the National Institute of Aging, National Institute of Nursing Research, and Patient‐Centered Outcomes Research Institute; he has received speaker honoraria from the Parkinson’s Foundation. Dr Applebaum receives financial support from the National Cancer Institute. Dr Paulsen receives support from the NINDS and NIBIB and has received personal compensation in the range of $0–$499 for serving on a Scientific Advisory or Data Safety Monitoring Board for Wave Life Sciences, has received personal compensation in the range of $0–$499 for serving on a Speakers Bureau for HDSA, and has received personal compensation in the range of $0–$499 for serving as a consultant with Acadia. Dr Bega has received personal compensation for consulting, serving on a scientific advisory board, speaking, or other activities with Speaker: Teva Pharmaceuticals, Acorda Therapeutics, Neurocrine Biosciences, Adamas Pharmaceuticals Consulting: Biogen Pharmaceuticals, Amgen Pharmaceuticals, Acadia Pharmaceuticals, Genentech, Inc, GE Healthcare, Gerson Lehrman Group, Guidepoint, L.E.K. C., and has received personal compensation in an editorial capacity for Editor: Annals of Clinical & Translational Neurology. Dr Frank has received personal compensation in the range of $500–$4999 for serving as a Consultant for Oscine Therapeutics. Dr Frank has received personal compensation in the range of $500–$4999 for serving as a Consultant for uniQure, has received personal compensation in the range of $500–$4999 for serving as a Consultant for MCG Health. The institution of Dr Frank has received research support from the Huntington’s Disease Society of America. The institution of Dr Frank has received research support from Michael J Fox Foundation. The institution of Dr Frank has received research support from Roche/Genentech. The institution of Dr Frank has received research support from CHDI Foundation. The institution of Dr Frank has received research support from the Huntington Study Group. The institution of Dr Frank has received research support from Triplet Therapeutics. Dr Hauser is supported or has received support from the Coleman Foundation, University of Florida, Instituto Nacional de Caˆncer, American Academy of Hospice and Palliative Medicine, Arnold P. Gold Foundation, National Institute on Aging, Seasons Hospice Foundation, Woodstock, HCSC Insurance Services Company, National Heart, Lung, and Blood Institute, Canadian Patient Safety Institute, Health Research, and Educational Trust, Icahn School of Medicine at Mount Sinai, Centers for Medicare and Medicaid Services, Agency for Healthcare Research and Quality, Children’s Hospitals and Clinics of Minnesota, Instituto Nacional de Caˆncer, Department of Veterans Affairs, National Center for Research Resources, NOVA Research Company, Instituto Nacional de Caˆncer, Lance Armstrong Foundation, Retirement Research Foundation, Society for the Arts in Healthcare, Medical College of Wisconsin, and the National Institute of Nursing Research. Dr Boileau reports no disclosures. Dr Depp receives support from the NIMH, VA, and NCATS. Dr Cella has research funding to his institution from NIH, Abbvie, Amgen, Bayer Healthcare, Bristol‐Myers Squibb, Clovis, Eli Lilly and Company, Glaxo Smith‐Kline, Johnson and Johnson, Novartis, Pfizer, and PledPharma; he has served as a consultant to Abbvie, Bristol‐Myers Squibb, Eli Lilly and Company, Johnson and Johnson, Novartis, and Pfizer. Dr Cella is the president of FACIT.org. Dr Carlozzi reports research grants from the NIH, the Neilsen Foundation, and CHDI, as well as contracts from Goldfinch, LLC, and Health and Human Services – Centers for Medicare & Medicaid Services; she receives honoraria for her role on the CHDI scientific advisory board and is a consultant on the TBI Congressionally mandated study.
